# Outcomes of patients with melanoma brain metastases treated with ipilimumab and nivolumab with or without upfront comprehensive stereotactic radiosurgery

**DOI:** 10.1093/noajnl/vdaf276

**Published:** 2026-01-07

**Authors:** Troy J Kleber, Denái R Milton, Subhiksha Srinivasan, Bikash Panthi, Warren Floyd, Eric A Goethe, Michael A Davies, Hussein A Tawbi, Isabella C Glitza Oliva, Diana Kaya, Jing Li, Todd A Swanson, Subha Perni, Martin C Tom, Chenyang Wang, Sujit Prabhu, Jeffrey S Weinberg, Ian E McCutcheon, Caroline Chung, Sherise D Ferguson, Thomas H Beckham

**Affiliations:** Department of Radiation Oncology, Division of Radiation Oncology, The University of Texas MD Anderson Cancer Center, Houston, Texas; Department of Biostatistics, The University of Texas MD Anderson Cancer Center, Houston, Texas; McGovern Medical School, The University of Texas Health Science Center, Houston, Texas; Department of CNS Radiation Oncology, Division of Radiation Oncology, The University of Texas MD Anderson Cancer Center, Houston, Texas; Department of Radiation Oncology, Division of Radiation Oncology, The University of Texas MD Anderson Cancer Center, Houston, Texas; Department of Neurosurgery, Baylor College of Medicine, Houston, Texas; Department of Melanoma Medical Oncology, Division of Cancer Medicine, The University of Texas MD Anderson Cancer Center, Houston, Texas; Department of Melanoma Medical Oncology, Division of Cancer Medicine, The University of Texas MD Anderson Cancer Center, Houston, Texas; Department of Melanoma Medical Oncology, Division of Cancer Medicine, The University of Texas MD Anderson Cancer Center, Houston, Texas; Department of Neuroradiology, Division of Diagnostic Imaging, The University of Texas MD Anderson Cancer Center, Houston, Texas; Department of CNS Radiation Oncology, Division of Radiation Oncology, The University of Texas MD Anderson Cancer Center, Houston, Texas; Department of CNS Radiation Oncology, Division of Radiation Oncology, The University of Texas MD Anderson Cancer Center, Houston, Texas; Department of CNS Radiation Oncology, Division of Radiation Oncology, The University of Texas MD Anderson Cancer Center, Houston, Texas; Department of CNS Radiation Oncology, Division of Radiation Oncology, The University of Texas MD Anderson Cancer Center, Houston, Texas; Department of CNS Radiation Oncology, Division of Radiation Oncology, The University of Texas MD Anderson Cancer Center, Houston, Texas; Department of Neurosurgery, Division of Surgery, The University of Texas MD Anderson Cancer Center, Houston, Texas; Department of Neurosurgery, Division of Surgery, The University of Texas MD Anderson Cancer Center, Houston, Texas; Department of Neurosurgery, Division of Surgery, The University of Texas MD Anderson Cancer Center, Houston, Texas; Department of CNS Radiation Oncology, Division of Radiation Oncology, The University of Texas MD Anderson Cancer Center, Houston, Texas; Department of Neurosurgery, Division of Surgery, The University of Texas MD Anderson Cancer Center, Houston, Texas; Department of CNS Radiation Oncology, Division of Radiation Oncology, The University of Texas MD Anderson Cancer Center, Houston, Texas

**Keywords:** brain metastasis, checkpoint inhibitor, immunotherapy, melanoma, stereotactic radiosurgery

## Abstract

**Background:**

The efficacy of ipilimumab and nivolumab (ipi/nivo) for melanoma brain metastases (MBMs) has been previously reported, leading to uncertainty regarding the optimal role of comprehensive stereotactic radiosurgery (cSRS). We therefore conducted a single-institution retrospective study to compare outcomes of upfront versus deferred cSRS for MBM treated with ipi/nivo.

**Methods:**

We identified patients who started ipi/nivo for newly diagnosed MBMs between 2018 and 2023, with or without upfront cSRS. Patients with >15 MBMs, leptomeningeal disease, or whole-brain radiotherapy at baseline were excluded. Outcomes were compared using multivariable regression and reported as adjusted hazard ratios (aHRs) with 95% CIs.

**Results:**

Of the 132 patients identified, 52.3% received upfront cSRS and 47.7% did not. Patients who received upfront cSRS had larger maximum MBMs (median 2.3 vs 0.7 cm; *P* < .001), more symptomatic MBMs (59.4% vs 11.1%; *P* < .001), higher rates of upfront craniotomy (47.8% vs 7.9%; *P* < .001), and fewer BRAF V600 mutations (34.8% vs 54.0%; *P* = .035). Upfront cSRS was not associated with longer overall survival (median 47.0 mo vs not reached; aHR = 1.01 [95% CI, 0.60-1.68]; *P* = .98) but was associated with reduced incidence of intracranial progression (median 37.6 vs 5.5 mo; aHR = 0.40 [95% CI, 0.25-0.64]; *P* < .001).

**Conclusions:**

In this retrospective study, upfront cSRS was more often used in patients with higher-risk MBM and was associated with improved intracranial control, although no significant survival benefit was observed. These findings suggest that starting ipi/nivo alone may be reasonable for lower-risk MBM, but prospective studies are needed to guide optimal integration of cSRS.

Key Points

- Combined ipilimumab and nivolumab is commonly used for melanoma brain metastases.
- Given intracranial efficacy of ipi/nivo, deferred stereotactic radiosurgery (SRS) may be a reasonable approach.
- Upfront SRS is associated with improved local control and progression-free survival.

Importance of the Study

Although a diagnosis of melanoma brain metastasis (MBM) was historically associated with a dismal prognosis and poor survival, advancements in systemic therapy options have drastically improved outcomes for these patients. The dual-agent checkpoint inhibitor regimen of ipilimumab and nivolumab has shown particular promise in terms of intracranial efficacy, leading to questions regarding the need for upfront intracranial radiation for patients started on this regimen. We therefore report our retrospective study investigating the outcomes of upfront versus deferred stereotactic radiosurgery (SRS) for patients with newly diagnosed MBM treated with ipilimumab and nivolumab. Our study revealed improved local control and progression-free survival from the use of upfront SRS. Overall survival did not differ between the 2 treatment approaches, however, suggesting that deferred SRS may be reasonable for patients with lower-risk MBM starting ipilimumab and nivolumab. Our study highlights the importance of multidisciplinary discussion for patients with MBM.

Melanoma brain metastases (MBMs), which affect 40%-50% of patients with melanoma during the course of their disease, are a major source of morbidity and mortality.^1^^,^^2^ Expected survival duration following a diagnosis of MBM was historically less than 6 months.^2^ However, the advancement of both local and systemic therapies has dramatically shifted the prognosis for patients,^3^^,^^4^ with median overall survival (OS) now estimated at 14.4 months since 2014.^3^

The dual-agent checkpoint inhibitor regimen of ipilimumab and nivolumab (ipi/nivo) has been shown to be particularly effective in stage IV melanoma patients, with recent results demonstrating 10-year OS of greater than 50% in patients without intracranial involvement.^5^ Ipilimumab and nivolumab has also demonstrated long-term benefit in patients with MBMs, with 2 separate clinical trials reporting intracranial clinical benefit rates of 57%.^6^^,^^7^

These findings raise an important clinical question regarding the optimal integration of stereotactic radiosurgery (SRS), the established standard approach for patients with limited brain metastases,^8^^,^^9^ with ipi/nivo in the management of MBMs. While prior clinical research has established the efficacy of ipi/nivo, it remains unclear whether upfront SRS confers additional benefit beyond systemic therapy alone or whether its use can be safely deferred in selected patients. To address this gap, we conducted a retrospective study to compare baseline characteristics and clinical outcomes between those who did and did not receive upfront SRS. We hypothesized that upfront SRS would be more commonly used in patients with higher-risk disease features and would be associated with improved intracranial control and OS when added to ipi/nivo.

## Methods

### Inclusion and Exclusion Criteria

Patients who started ipi/nivo for newly diagnosed MBM between January 1, 2018, and December 31, 2023, were identified from a single-institutional registry at The University of Texas MD Anderson Cancer Center (Houston, TX, United States). All patients were prescribed ipi/nivo at either standard dose (ipilimumab 3 mg/kg; nivolumab 1 mg/kg) or flipped dose (ipilimumab 1 mg/kg; nivolumab 3 mg/kg) every 3 weeks for 4 doses, followed by nivolumab maintenance therapy, with treatment termination or adjustments based on physician’s discretion. Patients were excluded if they had a diagnosis of leptomeningeal disease or more than 15 MBMs at baseline since these patients are generally considered ineligible for upfront SRS.^10^ Patients were also excluded if they received upfront whole-brain radiotherapy (WBRT), had a history of previously treated MBM, or had no post-treatment diagnostic brain imaging after starting ipi/nivo. Patients receiving any additional systemic therapy agents simultaneously with the start of ipi/nivo were also excluded. However, patients who initiated new systemic therapy subsequent to starting ipi/nivo (ie, after at least 1 follow-up scan) were not excluded. Institutional Review Board approval was obtained, and Strengthening the Reporting of Observation Studies in Epidemiology (STROBE) guidelines were followed in the design and reporting of this study.

### Data Collection

Patient characteristics, treatment details, and clinical outcomes were collected from electronic medical records. Patients were categorized based on whether they had received upfront comprehensive SRS (cSRS). “Upfront” indicated that SRS was initiated before or within 6 weeks after the start of ipi/nivo, provided there was no intracranial progression during that time interval. Patients who experienced intracranial progression after starting ipi/nivo but before SRS were included in the no upfront cSRS cohort, and this course of SRS was classified as salvage therapy. “Comprehensive” indicated that all baseline MBMs visualized radiographically and, if applicable, all post-operative cavities were treated with SRS. Patients who received SRS to some but not all of their baseline MBMs were included in the no upfront cSRS cohort.

Clinical outcomes included OS, progression-free survival (PFS), intracranial PFS (IC-PFS), extracranial PFS (EC-PFS), time to intracranial progression, time to start of next-line systemic therapy, and intracranial clinical benefit. Additionally, patients with intracranial progression were further categorized as local or distant intracranial progression based on their first instance of intracranial progression. Patients who died were further classified as neurologic or systemic death.

Post-treatment diagnostic imaging was reviewed to assess for intracranial progression according to criteria from the Response Assessment in Neuro-Oncology Brain Metastases.^11^ Intracranial clinical benefit was defined as complete response, partial response, or stable disease through 6 months for all MBMs, as reported previously.^6^ Local intracranial failure was defined as the radiographic progression of 1 or more MBMs that had been present at baseline. When available, advanced brain tumor imaging that incorporates sophisticated techniques for neuroimaging acquisition and interpretation was reviewed to distinguish tumor progression from radionecrosis for SRS-treated lesions.^9^^,^^12^ Intracranial hemorrhage without visible radiographic enlargement of the parenchymal lesion was not considered local failure. Distant intracranial failure was defined as the development of new intracranial disease. Extracranial progression was also assessed based on a review of all post-treatment systemic imaging and was defined per Response Evaluation Criteria in Solid Tumors version 1.1.^13^ Previously reported definitions for neurologic and systemic death were followed,^14^ with neurologic death defined as death in the context of progressive or severe neurologic dysfunction from intracranial disease, treatment-related adverse events, or intercurrent illness.

Treatment-related adverse events were also recorded. Radionecrosis was diagnosed radiographically, based on contrast-weighted magnetic resonance imaging and advanced brain tumor imaging (if available), or diagnosed pathologically by resection. Radionecrosis events were assessed for all MBMs targeted by upfront SRS and categorized according to the four-tier International Stereotactic Radiosurgery Society (ISRS) grading system.^15^ Events of clinically significant intracranial hemorrhage were defined as MBM-associated intracranial hemorrhage occurring within 90 days of treatment onset and requiring surgical evacuation.

### Statistical Analysis

OS, PFS, IC-PFS, and EC-PFS were estimated using the Kaplan-Meier method, and associations between these outcomes and covariates were determined using univariate and multivariable Cox proportional hazards regression models. When assessing associations between post-SRS symptomatic radionecrosis and outcomes, radionecrosis was included in the models as a time-dependent covariate. The cumulative incidences of intracranial progression and starting of next-line systemic therapy were estimated using the competing risks method, and associations between these outcomes and covariates were determined using univariate and multivariable proportional subdistribution hazards regression models.^16^ The competing risk included was death, and patients who did not experience the event and were still alive at their last follow-up date were censored. All clinical outcomes were computed from the date of ipi/nivo initiation.

Since the treatment groups were not randomized, we used stabilized inverse probability of treatment weighting (IPTW) in all multivariable regression models to correct for potential bias when making statistical comparisons between the 2 treatment cohorts.^17^ The logistic regression model that produced the propensity scores used to compute the stabilized IPTW included BRAF V600 mutation, maximum MBM size, symptomatic MBM, and receipt of upfront craniotomy. The IPTW multivariable regression models compared treatment groups with adjustment for BRAF V600 mutation, status of extracranial disease at MBM diagnosis, symptomatic MBM, receipt of upfront craniotomy, and synchronicity between MBM and metastasis diagnoses.

All statistical analyses were performed using SAS 9.4 for Windows (SAS Institute Inc., Cary, NC). All statistical tests used a significance level of 5%. All hazard ratios (HRs) and adjusted HRs (aHRs) are reported with 95% CIs. No adjustments for multiple testing were made.

## Results

We identified 132 patients with 476 total MBMs and a median follow-up time of 26.4 months. A flow diagram for inclusion and exclusion criteria is included in Figure S1. Median age was 60 years; 67.4% (*n* = 89) were male; 90.2% (*n* = 119) had an Eastern Cooperative Oncology Group performance status of 0 or 1; 20.5% (*n* = 27) had a history of systemic therapy for metastatic melanoma prior to their MBM diagnosis; and 43.9% (*n* = 58) had a BRAF V600 mutation, including V600E (*n* = 46), V600K (*n* = 10), and V600R (*n* = 2) mutations. In terms of the ipi/nivo prescription, 89.4% (*n* = 118) received standard dose, and 10.6% (n = 14) received flipped dose. Additionally, 36.4% (*n* = 48) had symptomatic MBM, including 9 patients who remained on dexamethasone through the start of ipi/nivo. Median OS for all patients was 47.0 months (95% CI, 33.1 mo—not reached [NR]).

Of the 132 patients, 52.3% (*n* = 69) received upfront cSRS, and 47.7% (*n* = 63) did not. Baseline patient characteristics for each treatment cohort are displayed in Table 1. Among the patients who received upfront cSRS, 47.8% (*n* = 33) also underwent upfront craniotomy for MBM resection. Among the patients who did not receive upfront cSRS, 7.9% (*n* = 5) underwent upfront craniotomy, and 4.8% (*n* = 3) received upfront SRS to some but not all of their intracranial lesions.

**Table 1.** Baseline characteristics of patients who received ipi/nivo with or without upfront cSRS for newly diagnosed MBM

Characteristic	All patients (*n* = 132)	Received upfront cSRS (*n* = 69)	Did not receive upfront cSRS (*n* = 63)	*P* ^d^
Age, years				
Median	61	62	60	.30
IQR	51-67	51-69	51-66	
Range	21-88	28-88	21-82	
Sex, *n* (%)				
Female	43 (32.6)	22 (31.9)	21 (33.3)	1.00
Male	89 (67.4)	47 (68.1)	42 (66.7)	
ECOG performance status, *n* (%)				
0-1	119 (90.2)	63 (91.3)	56 (88.9)	.77
2-3	13 (9.8)	6 (8.7)	7 (11.1)	
BRAF V600 mutation, n (%)				
Yes	58 (43.9)	24 (34.8)	34 (54.0)	.04
No or unknown	74 (56.1)	45 (65.2)	29 (46.0)	
LDH at start of ipi/nivo, *n* (%)				
>250 U/L	59 (50.4)	31 (53.4)	28 (47.5)	.58
≤250 U/L	58 (49.6)	27 (46.6)	31 (52.5)	
Unknown	15	11	4	
Synchronicity between MBM and metastasis diagnoses, *n* (%)				
Synchronous	105 (79.5)	54 (78.3)	51 (81.0)	.83
Metachronous^a^	27 (20.5)	15 (21.7)	12 (19.0)	
History of checkpoint inhibitor, *n* (%)				
Yes^b^	35 (26.5)	19 (27.5)	16 (25.4)	.84
No	97 (73.5)	50 (72.5)	47 (74.6)	
History of BRAF/MEK inhibitor, *n* (%)				
Yes^b^	12 (9.1)	5 (7.2)	7 (11.1)	.55
No	120 (90.9)	64 (92.8)	56 (88.9)	
Status of extracranial disease at MBM diagnosis, *n* (%)				
Present, uncontrolled	108 (81.8)	53 (76.8)	55 (87.3)	.07
Present, controlled	9 (6.8)	4 (5.8)	5 (7.9)	
Absent	15 (11.4)	12 (17.4)	3 (4.8)	
Total number of MBM				
Median	2	2	2	.19
IQR	1-5	1-4	1-6	
Range	1-15	1-15	1-15	
Maximum size of MBM,^c^ cm				
Median	1.4	2.3	0.7	<.001
IQR	0.6-2.7	1.4-3.2	0.5-1.2	
Range	0.5-5.4	0.5-6.1	0.3-5.4	
Symptomatic MBM, *n* (%)				
Yes	48 (36.4)	41 (59.4)	7 (11.1)	<.001
No	84 (63.6)	28 (40.6)	56 (88.9)	
Received upfront surgery for MBM, *n* (%)				
Yes	38 (28.8)	33 (47.8)	5 (7.9)	<.001
No	94 (71.2)	36 (52.2)	58 (92.1)	
Received upfront SRS for MBM, *n* (%)				
Yes, to all MBM	69 (52.3)	69 (100.0)	0 (0)	NA
Yes, to some MBM	3 (2.3)	0 (0)	3 (4.8)	
No	60 (45.5)	0 (0)	60 (95.2)	
Ipi/nivo dose				
Standard dose	118 (89.4)	59 (85.5)	59 (93.7)	.13
Flipped dose	14 (10.6)	10 (14.5)	4 (6.3)	
Number of cycles of ipi/nivo or nivo monotherapy completed before starting next-line systemic therapy, *n* (%)				
1	14 (10.6)	9 (13.0)	5 (7.9)	.30
2	26 (19.7)	10 (14.5)	16 (25.4)	
3	23 (17.4)	12 (17.4)	11 (17.5)	
4	9 (6.8)	3 (4.3)	6 (9.5)	
≥5	60 (45.5)	35 (50.7)	25 (39.7)	
Follow-up time from initiation of ipi/nivo, months				
Median	26.4	26.4	26.3	.60
IQR	11.3-46.7	11.2-41.4	11.3-47.6	
Range	1.1-78.6	1.1-78.6	1.9-77.9	

Abbreviations: cSRS, comprehensive SRS; ECOG, Eastern Cooperative Oncology Group; ipi/nivo, ipilimumab and nivolumab; IQR, interquartile range; LDH, lactate dehydrogenase; MBM, melanoma brain metastasis; NA, not applicable; SRS, stereotactic radiosurgery.

a Metachronous indicates that the patient was previously diagnosed with metastatic melanoma without brain metastases and started a prior line of systemic therapy before having disease progression with brain metastases.

b In either the curative or metastatic setting.

c For MBM resected by upfront craniotomy, only the pre-operative size was considered; post-operative cavity sizes were not recorded.

d Comparisons between the 2 cohorts were conducted using Fisher’s exact test for categorical variables and Wilcoxon rank-sum test for continuous variables.

Compared with those who did not receive upfront cSRS, patients who received upfront cSRS had a larger maximum MBM size (median 2.3 vs 0.7 cm; *P* < .001) and a higher rate of symptomatic brain metastases (59.4% vs 11.1%; *P* < .001). Additionally, BRAF V600 mutations were less common among patients who received cSRS than among those who did not (34.8% vs 54.0%; *P* = .035).

Associations between baseline patient characteristics and clinical outcomes on univariate analyses are shown in Table 2. Of note, a metachronous diagnosis of MBM, a history of ­checkpoint inhibitors, and an increased total number of MBM were associated with decreased OS and IC-PFS. Conversely, treatment with upfront craniotomy was associated with improved OS and IC-PFS on univariate analyses.

**Table 2.** Associations between baseline characteristics and clinical outcomes on univariate analyses

Characteristic	Comparison	OS HR (95% CI)	*P*	IC-PFS HR (95% CI)	*P*
Synchronicity for MBM diagnosis	Metachronous^a^ vs synchronous	2.32 (1.32-4.10)	.004	2.35 (1.44-3.82)	<.001
History of checkpoint inhibitor^b^	Yes vs no	1.87 (1.09-3.23)	.023	1.92 (1.21-3.04)	.006
History of BRAF/MEK inhibitor^b^	Yes vs no	1.57 (0.71-3.45)	.27	2.09 (1.07-4.05)	.030
BRAF V600 status	Mutant vs wild-type/unknown	1.09 (0.65-1.83)	.74	1.62 (1.05-2.49)	.029
LDH at start of ipi/nivo	>250 vs ≤250/unknown	1.47 (0.88-2.47)	.14	0.84 (0.54-1.29)	.43
Status of extracranial disease at MBM diagnosis	Uncontrolled vs controlled/absent	0.75 (0.41-1.40)	.37	0.65 (0.38-1.09)	.10
Symptomatic MBM	Yes vs no	1.05 (0.62-1.78)	.86	1.03 (0.66-1.59)	.91
Received surgery upfront for MBM	Yes vs no	0.52 (0.28-0.99)	.045	0.59 (0.36-0.98)	.040
ECOG performance status	0-1 vs 2-3	0.63 (0.29-1.40)	.26	1.41 (0.73-2.75)	.31
Age	Continuous	1.03 (1.00-1.05)	.022	1.00 (0.98-1.01)	.71
Maximum size of MBM	Continuous	1.02 (0.86-1.23)	.79	0.93 (0.80-1.09)	.39
Total number of MBM	Continuous	1.08 (1.01-1.15)	.016	1.07 (1.02-1.12)	.009

Abbreviations: LDH, lactate dehydrogenase; ECOG, Eastern Cooperative Oncology Group; HR, hazard ratio; IC-PFS, intracranial progression-free survival; MBM, melanoma brain metastasis; OS, overall survival.

a Metachronous indicates that the patient was previously diagnosed with metastatic melanoma without brain metastases and started a prior line of systemic therapy before having disease progression with brain metastases.

b In either the curative or metastatic setting.

**Figure 1.**
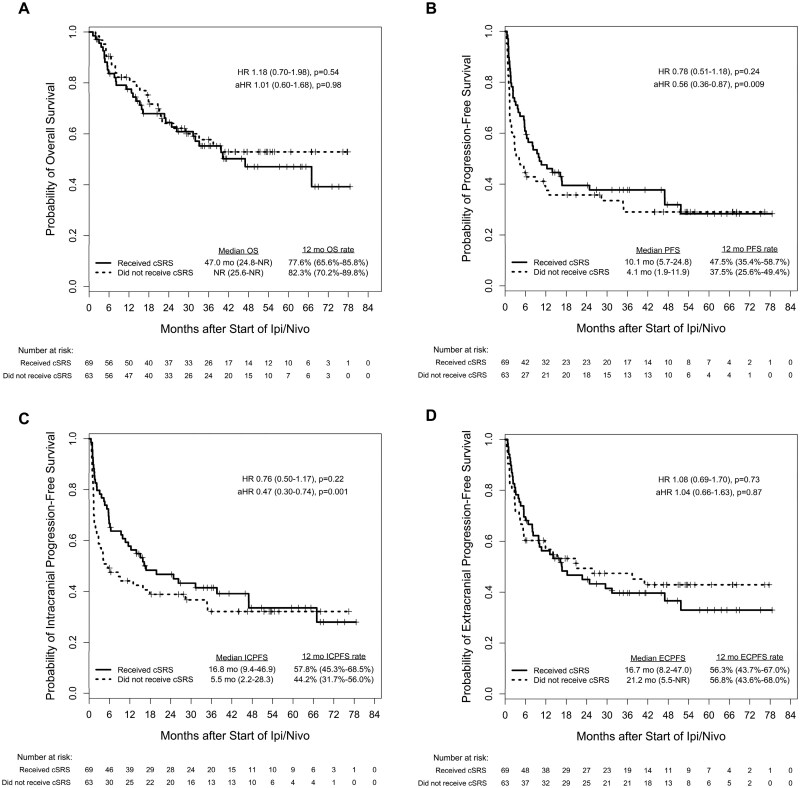
Kaplan-Meier curves for overall survival (A), progression-free survival (B), intracranial progression-free survival (C), and extracranial progression-free survival (D) by receipt of upfront cSRS among patients with melanoma brain metastases started on ipi/nivo. All estimates include 95% CIs in parentheses. aHR, adjusted HR; cSRS, comprehensive stereotactic radiosurgery; ECPFS, extracranial progression-free survival; HR, hazard ratio; ICPFS, intracranial progression-free survival; ipi/nivo, ipilimumab and nivolumab; NR, not reached; OS, overall survival; PFS, progression-free survival.

**Figure 2.**
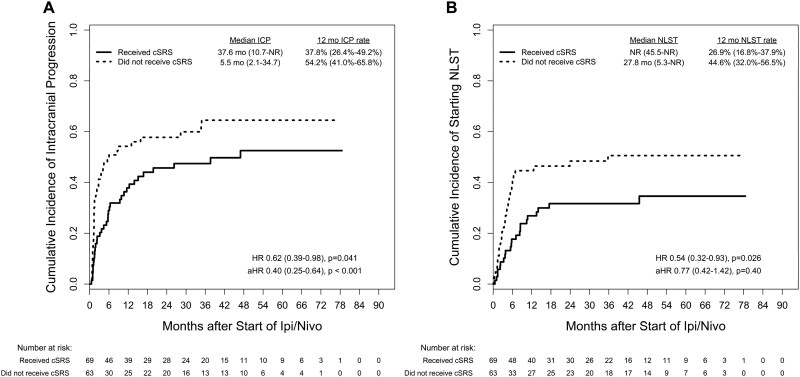
Associations between upfront cSRS and the cumulative incidence of intracranial progression (A) and the starting of next-line systemic therapy (B) among patients with melanoma brain metastases treated with ipi/nivo. All estimates include 95% CIs in parentheses. aHR, adjusted HR; cSRS, comprehensive stereotactic radiosurgery; HR, hazard ratio; ICP, intracranial progression; ipi/nivo, ipilimumab and nivolumab; NLST, next-line systemic therapy; NR, not reached.

Clinical outcomes between treatment cohorts are shown in Figure 1. In terms of OS, there was no significant difference between those who received upfront cSRS compared to those who did not (median 47.0 mo vs NR; aHR = 1.01 [95% CI, 0.60-1.68]; *P* = .98). However, on multivariable analyses, upfront cSRS was associated with significant improvement in both IC-PFS (median 16.8 vs 5.5 mo; aHR = 0.47 [95% CI, 0.30-0.74]; *P* = .001) and overall PFS (median 10.1 vs 4.1 mo; aHR = 0.56 [95% CI, 0.36-0.87]; *P* = .009). EC-PFS was not significantly different between treatment cohorts (median 16.7 vs 21.2 mo; aHR 1.04 [95% CI, 0.66-1.63]; *P* = .87). Incidence of neurologic death was also not significantly different between treatment cohorts (14.5% vs 17.5%; *P* = .64; Table S1). The cumulative incidences of intracranial progression and the starting of next-line systemic therapy are shown in Figure 2. Upfront cSRS was associated with significantly longer time to intracranial progression (median 37.6 vs 5.5 mo; aHR = 0.40 [95% CI, 0.25-0.64]; *P* < .001). However, the difference in time to next-line systemic therapy was not statistically significant on multivariable analysis (median NR vs 27.8 mo; aHR = 0.77 [95% CI, 0.42-1.42]; *P* = .40). Intracranial clinical benefit occurred in 63.8% of patients who received cSRS and 46.0% of patients who did not.

**Figure 3.**
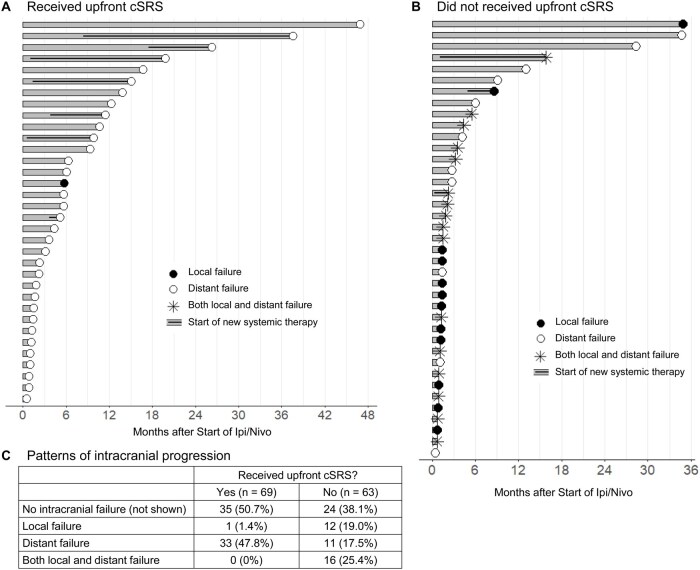
Patterns of intracranial progression among patients with melanoma brain metastasis started on ipi/nivo with (A) or without (B) upfront cSRS. Only the first instance of intracranial progression after starting ipi/nivo is presented. The start of a new systemic therapy regimen following ipi/nivo is represented by a thick horizontal black line within the bar. Counts and percentages for each intracranial progression pattern are outlined in the table (C). cSRS, comprehensive stereotactic radiosurgery; ipi/nivo, ipilimumab and nivolumab.

Swimmer plots representing the first instance of intracranial progression after starting ipi/nivo with or without cSRS are displayed in Figure 3. Local intracranial failure occurred in 1.4% of patients who received upfront cSRS and 44.4% of patients who did not. Distant intracranial failure occurred in 47.8% of patients who received cSRS and 42.9% of patients who did not. Salvage therapies received after the first instance of intracranial progression are shown in Table 3. Salvage local intracranial therapy was received by 42.0% (*n* = 29) of patients treated with upfront cSRS and 58.7% (*n* = 37) of patients treated without upfront cSRS. Patterns of salvage therapy between treatment cohorts did not differ significantly between cohorts, with most patients receiving SRS for salvage. For patients who experienced intracranial progression, median OS duration after their first instance of intracranial progression was 14.1 months (95% CI, 7.8-19.8 mo).

**Table 3.** Salvage therapies received after the first instance of intracranial progression

Salvage therapy	All patients (*n* = 73)	Received upfront cSRS (*n* = 34)	Did not receive upfront cSRS (*n* = 39)	*P*
SRS	41 (56.2%)	18 (52.9%)	23 (59.0%)	.46
WBRT	13 (17.8%)	5 (14.7%)	8 (20.5%)	
Craniotomy^a^	8 (11.0%)	5 (14.7%)	3 (7.7%)	
BRAF and MEK inhibitor without local intracranial therapy	6 (8.2%)	2 (5.9%)^b^	4 (10.3%)^c^	
Lost to follow-up or death	5 (6.8%)	4 (11.8%)	1 (2.6%)	

Abbreviations: cSRS, comprehensive SRS; SRS, stereotactic radiosurgery; WBRT, whole-brain radiotherapy.

a These patients subsequently proceeded with post-operative SRS, as appropriate.

b Of these 2 patients, 1 later received salvage intracranial radiation after progressing on the BRAF/MEK inhibitor regimen.

c Of these 4 patients, 3 later received salvage intracranial radiation after progressing on the BRAF/MEK inhibitor regimen.

The cumulative incidences of symptomatic radionecrosis for both treatment cohorts are shown in Figure S2, which includes radionecrosis resulting from upfront or salvage SRS courses. Among patients treated with upfront cSRS, rates of symptomatic radionecrosis at 1 and 2 years are 14.8% (95% CI, 4.8%-23.8%) and 24.2% (95% CI, 11.0%-35.4%), respectively. Among patients treated without upfront cSRS, rates of symptomatic radionecrosis at 1 and 2 years are 2.0% (95% CI, 0.0%-5.8%) and 4.2% (95% CI, 0.0%-9.8%), respectively.

Post-treatment symptomatic radionecrosis was also assessed specifically for the 72 patients who received upfront SRS, either comprehensively (*n* = 69) or partially (*n* = 3), to a total of 224 intracranial lesions. Of these patients, 18.1% (*n* = 13) developed symptomatic radionecrosis, involving a total of 5.8% (*n* = 13) of the lesions. Five patients experienced ISRS grade 2 radionecrosis, 5 patients experienced ISRS grade 3 radionecrosis, and 3 patients experienced ISRS grade 4 radionecrosis. The median time to radionecrosis presentation after completing upfront SRS was 10.9 months (range, 5.7-38.4 months). Patients treated with upfront SRS with subsequent symptomatic radionecrosis had significantly improved IC-PFS compared to those treated with upfront SRS without symptomatic radionecrosis (HR = 0.20 [95% CI, 0.06-0.66]; *P* = .008) and to those treated without upfront SRS (HR = 0.23 [95% CI, 0.07-0.73]; *P* = .013). Additionally, these patients had improved OS compared both to those treated with upfront SRS without symptomatic radionecrosis (HR = 0.33 [95% CI, 0.10-1.08]; *P* = .07) and to those treated without upfront SRS (HR = 0.48 [95% CI, 0.14-1.58]; *P* = .22), although these OS improvements were not statistically significant.

Two events of clinically significant intracranial hemorrhage from an irradiated lesion were noted at 42 days and 78 days after SRS, respectively. One event of clinically significant intracranial hemorrhage from an unirradiated lesion was noted at 60 days after ipi/nivo initiation.

## Discussion

Our retrospective study yields insight into the role of upfront cSRS for patients receiving ipi/nivo for newly diagnosed MBMs. Notably, life expectancy was generally favorable, with a median OS of 47 months for all patients in our study, and there was no significant difference in OS between those treated with or without upfront cSRS. We also found that upfront cSRS was associated with a significant reduction in intracranial progression despite this cohort having higher-risk MBM features. The median IC-PFS duration was 16.8 months for patients who received cSRS and 5.5 months for patients who did not, and intracranial clinical benefit occurred in 64% and 46%, respectively.

The advantage of upfront SRS appears to stem primarily from its ability to provide durable local control. In our study, only 1.4% of patients treated with upfront cSRS experienced local failure, compared to 44.4% of patients treated without upfront cSRS, and this translated into a significant improvement in both IC-PFS and overall PFS. In contrast, upfront cSRS did not noticeably affect disease control at non-targeted sites. Rates of distant intracranial failure were similar between cohorts, occurring in 47.8% of patients treated with upfront cSRS and 42.9% of those treated without upfront cSRS. EC-PFS was also similar between the treatment groups. This is consistent with previous randomized studies, which have been unable to reliably demonstrate a clinically evident abscopal effect of ablative radiation in combination with immunotherapy.^18–21^

Other retrospective studies have also explored the benefits of upfront local intracranial therapy in patients receiving ipi/nivo. Mandalà et al.^2^ and Amaral et al.^23^, who investigated multi-institutional cohorts of patients with MBM treated with ipi/nivo, identified several factors associated with improved OS, including the use of local intracranial therapy. These findings contrast somewhat with those of our own study, which did not show a significant survival benefit from upfront cSRS. However, a direct comparison between the studies is difficult owing to differences in sample sizes, selection criteria, treatment categorizations, and methods for mitigating immortal time bias. Rather, our results align more closely with those of Tang et al.^24^, who similarly reported a single-institution retrospective cohort of MBM treated with ipi/nivo with or without upfront SRS, observing significant improvements from SRS in terms of local control but not OS.

The use of ipi/nivo alone for MBM became increasingly prevalent after the findings of Tawbi et al.^6^ and Long et al.^7^ Both trials reported an intracranial clinical benefit rate of 57% from ipi/nivo,^6^^,^^7^ which was notably superior to the 20%-30% rates observed in trials of single-agent checkpoint inhibitors for MBM.^7^^, ^^25–27^ In our study, the intracranial clinical benefit rate of ipi/nivo alone for MBM was 46%, further supporting the intracranial efficacy of dual-agent checkpoint inhibitors in this real-world cohort. Additionally, our study demonstrated a higher intracranial clinical benefit rate of 64% for ipi/nivo combined with upfront cSRS. However, prospective randomized trials, such as the ongoing ABC-X study (NCT03340129), are essential to more definitively evaluate the potential benefits of SRS in this setting.

Imbalances between treatment cohorts are inevitable in retrospective studies, and our findings must be interpreted with this in mind. At our institution, the use of upfront cSRS was understandably more common in higher-risk patients, including those who had larger or symptomatic brain metastases or who underwent upfront craniotomy. Moreover, BRAF V600 mutations, which allow for targeted therapy as a salvage option and consequently have been associated with improved outcomes,^4^ were more common in patients treated with ipi/nivo without upfront cSRS. Although we accounted for these differences by performing a stabilized IPTW multivariable analysis, unmeasured confounders may still exist. Therefore, our finding that patients who received upfront cSRS had similar OS compared with the lower-risk patients who did not receive upfront cSRS may be a testament to the value of SRS in the high-risk patient population.

Our observation that upfront cSRS was associated with improved PFS but similar OS in our study may also be partly explained by the effectiveness of salvage therapy options for intracranial progression. In both cohorts, most patients received SRS after their first instance of intracranial progression, and the use of WBRT as salvage therapy was the next most common. These well-utilized salvage approaches likely contributed to the relatively long median OS duration of 14.1 months after intracranial progression.

When considering upfront SRS for patients with MBM starting ipi/nivo, the benefits of SRS in terms of preventing intracranial progression must be weighed against the increased commitment, cost, and toxicity concerns of this additional therapy. The risk of radionecrosis must also be considered, particularly for this population receiving immune-mediating therapies. Prior studies demonstrated that the incidence of radionecrosis increases when SRS is delivered concurrently with immunotherapy,^28–31^ possibly correlating with PD-L1 expression.^32^ This substantiates the theory that checkpoint blockade can exacerbate the intracranial inflammatory response to SRS. Interestingly, radionecrosis has also been shown to correlate with improved intracranial control,^33^ which was corroborated within our study and may be evidence of a synergistic inflammatory response from the combination of SRS and immunotherapy. An added complexity is the potential need for corticosteroids for radionecrosis, which could impact the efficacy of immunotherapy, although clinical studies attempting to elucidate this concern have yielded mixed results.^34–37^ Fortunately, rates of symptomatic radionecrosis from SRS are relatively low. In our study, 18.1% of patients experienced symptomatic radionecrosis related to their upfront SRS course. This incidence is consistent with previously reported rates of 18%-20% among patients receiving combined SRS and immunotherapy.^28^^,^^30^

Intracranial hemorrhage is also commonly cited as a concerning toxicity from SRS,^9^ though MBMs have a strong propensity for hemorrhage even without treatment.^38–40^ Fortunately, intracranial hemorrhage was rare in our patient population, occurring in only 2 irradiated lesions and 1 unirradiated lesion after treatment was started. This is particularly reassuring given that the patients treated with SRS in our study tended to have larger or symptomatic lesions at baseline.

Our results must be interpreted in the context of the study’s multiple limitations. Given the retrospective nature of this research, our findings are subject to potential confounders that cannot be completely eliminated by multivariable analyses. In addition, any study comparing multimodality with single-modality treatment approaches is susceptible to immortal time bias. However, we minimized this bias by excluding patients who had no follow-up brain imaging after starting ipi/nivo, by measuring clinical outcomes from the time of ipi/nivo initiation instead of MBM diagnosis, and by providing a clear definition for upfront cSRS. Lastly, this study was based on a single-institutional registry, which may limit the generalizability of its findings.

In conclusion, ipi/nivo is an effective treatment for patients with newly diagnosed MBM. The use of upfront cSRS in these patients appears to decrease the likelihood of intracranial progression and mitigate high-risk features, such as the presence of large, symptomatic lesions, but does not appear to improve OS. These findings suggest that patients with lower-risk MBM may be reasonably observed on ipi/nivo alone, though close surveillance is critical since intracranial progression is commonly observed after a short interval. This study provides insights that may inform multidisciplinary discussions. However, given the retrospective, single-institution design of this study, prospective validation is necessary before drawing firm conclusions.

## Supplementary Material

vdaf276_Supplementary_Data

## Data Availability

Research data are stored in an institutional repository and will be shared upon reasonable request to the corresponding author following publication of the manuscript.

## References

[1] Patel JK , Didolkar MS, Pickren JW, Moore RH. Metastatic pattern of malignant melanoma. A study of 216 autopsy cases. Am J Surg. 1978;135:807-810. 665907 10.1016/0002-9610(78)90171-x

[2] Davies MA , Liu P, McIntyre S, et al. Prognostic factors for survival in melanoma patients with brain metastases. Cancer. 2011;117:1687-1696. 20960525 10.1002/cncr.25634

[3] Hasanov M , Milton DR, Davies AB, et al. Changes in outcomes and factors associated with survival in melanoma patients with brain metastases. Neuro Oncol. 2023;25:1310-1320. 36510640 10.1093/neuonc/noac251 PMC10326492

[4] Sperduto PW , Jiang W, Brown PD, et al. Estimating survival in melanoma patients with brain metastases: an update of the graded prognostic assessment for melanoma using molecular markers (melanoma-molGPA). Int J Radiat Oncol Biol Phys. 2017;99:812-816. 29063850 10.1016/j.ijrobp.2017.06.2454 PMC6925529

[5] Wolchok JD , Chiarion-Sileni V, Rutkowski P, et al. Final, 10-year outcomes with nivolumab plus ipilimumab in advanced melanoma. N Engl J Med. 2025;392:11-22. 39282897 10.1056/NEJMoa2407417 PMC12080919

[6] Tawbi HA , Forsyth PA, Algazi A, et al. Combined nivolumab and ipilimumab in melanoma metastatic to the brain. N Engl J Med. 2018; 379:722-730. 30134131 10.1056/NEJMoa1805453 PMC8011001

[7] Long GV , Atkinson V, Lo S, et al. Combination nivolumab and ipilimumab or nivolumab alone in melanoma brain metastases: a multicentre randomised phase 2 study. Lancet Oncol. 2018;19:672-681. 29602646 10.1016/S1470-2045(18)30139-6

[8] Yamamoto M , Serizawa T, Shuto T, et al. Stereotactic radiosurgery for patients with multiple brain metastases (JLGK0901): a multi-institutional prospective observational study. Lancet Oncol. 2014;15:387-395. 24621620 10.1016/S1470-2045(14)70061-0

[9] Ene CI , Abi Faraj C, Beckham TH, et al. Response of treatment-naive brain metastases to stereotactic radiosurgery. Nat Commun. 2024;15:3728. 38697991 10.1038/s41467-024-47998-8 PMC11066027

[10] Ladbury C , Pennock M, Yilmaz T, et al. Stereotactic radiosurgery in the management of brain metastases: a case-based radiosurgery society practice guideline. Adv Radiat Oncol. 2024;9:101402. 38292892 10.1016/j.adro.2023.101402 PMC10823095

[11] Lin NU , Lee EQ, Aoyama H, et al. Response assessment criteria for brain metastases: proposal from the RANO group. Lancet Oncol. 2015;16:e270-e278. 26065612 10.1016/S1470-2045(15)70057-4

[12] Dagher SA , Liu HL, Ozkara BB, et al. The impact of MRI-based advanced neuroimaging on neurooncologists’ clinical decision-making in patients with posttreatment high-grade glioma: a prospective survey-based study. AJR Am J Roentgenol. 2024;223:e2431595. 39140632 10.2214/AJR.24.31595

[13] Eisenhauer EA , Therasse P, Bogaerts J, et al. New response evaluation criteria in solid tumours: revised RECIST guideline (version 1.1). Eur J Cancer. 2009;45:228-247. 19097774 10.1016/j.ejca.2008.10.026

[14] Patchell RA , Tibbs PA, Regine WF, et al. Postoperative radiotherapy in the treatment of single metastases to the brain: a randomized trial. JAMA. 1998;280:1485-1489. 9809728 10.1001/jama.280.17.1485

[15] Vellayappan B , Lim-Fat MJ, Kotecha R, et al. A systematic review informing the management of symptomatic brain radiation necrosis after stereotactic radiosurgery and international stereotactic radiosurgery society recommendations. Int J Radiat Oncol Biol Phys. 2024;118:14-28. 37482137 10.1016/j.ijrobp.2023.07.015

[16] Fine JP , Gray RJ. A proportional hazards model for the subdistribution of a competing risk. J Am Stat Assoc. 1999;94:496-509.

[17] Robins JM , Hernán MA, Brumback B. Marginal structural models and causal inference in epidemiology. Epidemiology. 2000;11:550-560. 10955408 10.1097/00001648-200009000-00011

[18] Welsh J , Menon H, Chen D, et al. Pembrolizumab with or without radiation therapy for metastatic non-small cell lung cancer: A randomized phase I/II trial. J Immunother Cancer. 2020;8: e001001. 33051340 10.1136/jitc-2020-001001 PMC7555111

[19] McBride S , Sherman E, Tsai CJ, et al. Randomized phase II trial of nivolumab with stereotactic body radiotherapy versus nivolumab alone in metastatic head and neck squamous cell carcinoma. J Clin Oncol. 2021;39:30-37. 32822275 10.1200/JCO.20.00290 PMC8462641

[20] Theelen W , Peulen HMU, Lalezari F, et al. Effect of pembrolizumab after stereotactic body radiotherapy vs pembrolizumab alone on tumor response in patients with advanced non-small cell lung cancer: results of the PEMBRO-RT phase 2 randomized clinical trial. JAMA Oncol. 2019;5:1276-1282. 31294749 10.1001/jamaoncol.2019.1478 PMC6624814

[21] Kim S , Wuthrick E, Blakaj D, et al. Combined nivolumab and ipilimumab with or without stereotactic body radiation therapy for advanced Merkel cell carcinoma: a randomised, open label, phase 2 trial. Lancet. 2022;400:1008-1019. 36108657 10.1016/S0140-6736(22)01659-2 PMC9533323

[22] Mandalà M , Lorigan P, Sergi MC, et al. Combined immunotherapy in melanoma patients with brain metastases: a multicenter international study. Eur J Cancer. 2024;199:113542. 38266540 10.1016/j.ejca.2024.113542

[23] Amaral T , Kiecker F, Schaefer S, et al. Combined immunotherapy with nivolumab and ipilimumab with and without local therapy in patients with melanoma brain metastasis: a DeCOG study in 380 patients. J Immunother Cancer. 2020;8:e000333. 10.1136/jitc-2019-000333 PMC7206917 32221017

[24] Tang JD , Mills MN, Nakashima J, et al. Clinical outcomes of melanoma brain metastases treated with nivolumab and ipilimumab alone versus nivolumab and ipilimumab with stereotactic radiosurgery. J Neurooncol. 2024;166:431-440. 38310157 10.1007/s11060-023-04543-9

[25] Margolin K , Ernstoff MS, Hamid O, et al. Ipilimumab in patients with melanoma and brain metastases: an open-label, phase 2 trial. Lancet Oncol. 2012;13:459-465. 22456429 10.1016/S1470-2045(12)70090-6

[26] Kluger HM , Chiang V, Mahajan A, et al. Long-term survival of patients with melanoma with active brain metastases treated with pembrolizumab on a phase II trial. J Clin Oncol. 2019;37:52-60. 30407895 10.1200/JCO.18.00204 PMC6354772

[27] Qian JM , Yu JB, Mahajan A, Goldberg SB, Kluger HM, Chiang VLS. Frequent use of local therapy underscores need for multidisciplinary care in the management of patients with melanoma brain metastases treated with PD-1 inhibitors. Int J Radiat Oncol Biol Phys. 2019;105:1113-1118. 31479702 10.1016/j.ijrobp.2019.08.053

[28] Martin AM , Cagney DN, Catalano PJ, et al. Immunotherapy and symptomatic radiation necrosis in patients with brain metastases treated with stereotactic radiation. JAMA Oncol. 2018;4:1123-1124. 29327059 10.1001/jamaoncol.2017.3993 PMC5885198

[29] Colaco RJ , Martin P, Kluger HM, Yu JB, Chiang VL. Does immunotherapy increase the rate of radiation necrosis after radiosurgical treatment of brain metastases? J Neurosurg. 2016;125:17-23. 26544782 10.3171/2015.6.JNS142763

[30] Vaios EJ , Shenker RF, Hendrickson PG, et al. Symptomatic necrosis with dual immune-checkpoint inhibition and radiosurgery for brain metastases. JAMA Network Open. 2025;8:e254347. 40202761 10.1001/jamanetworkopen.2025.4347 PMC11983232

[31] Helis CA , Hughes RT, Glenn CW, et al. Predictors of adverse radiation effect in brain metastasis patients treated with stereotactic radiosurgery and immune checkpoint inhibitor therapy. Int J Radiat Oncol Biol Phys. 2020;108:295-303. 32615262 10.1016/j.ijrobp.2020.06.057

[32] Adib E , Nassar AH, Bou Farhat E, et al. PD-L1, tumor mutational burden, and outcomes in non-small cell lung cancer with brain metastases: a brief report. JTO Clin Res Rep. 2025;6:100797. 10.1016/j.jtocrr.2025.100797 PMC11960624 40170983

[33] Hall J , Lui K, Tan X, et al. Factors associated with radiation necrosis and intracranial control in patients treated with immune checkpoint inhibitors and stereotactic radiotherapy. Radiother Oncol. 2023;189:109920. 37769968 10.1016/j.radonc.2023.109920

[34] Ricciuti B , Dahlberg SE, Adeni A, Sholl LM, Nishino M, Awad MM. Immune checkpoint inhibitor outcomes for patients with non-small-cell lung cancer receiving baseline corticosteroids for palliative versus nonpalliative indications. J Clin Oncol. 2019;37:1927-1934. 31206316 10.1200/JCO.19.00189

[35] De Giglio A , Mezquita L, Auclin E, et al. Impact of intercurrent introduction of steroids on clinical outcomes in advanced non-small-cell lung cancer (NSCLC) patients under immune-checkpoint inhibitors (ICI). Cancers (Basel). 2020;12:2827. 33007977 10.3390/cancers12102827 PMC7599488

[36] Petrelli F , Signorelli D, Ghidini M, et al. Association of steroids use with survival in patients treated with immune checkpoint inhibitors: a systematic review and meta-analysis. Cancers (Basel *).* 2020;12:546. 32120803 10.3390/cancers12030546 PMC7139305

[37] Weber JS , Hodi FS, Wolchok JD, et al. Safety profile of nivolumab monotherapy: a pooled analysis of patients with advanced melanoma. J Clin Oncol. 2017;35:785-792. 28068177 10.1200/JCO.2015.66.1389

[38] Xia Y , Mashouf LA, Maxwell R, et al. Adjuvant radiotherapy and outcomes of presumed hemorrhagic melanoma brain metastases without malignant cells. Surg Neurol Int. 2018;9:146. 30105140 10.4103/sni.sni_140_18 PMC6080145

[39] Kondziolka D , Bernstein M, Resch L, et al. Significance of hemorrhage into brain tumors: clinicopathological study. J Neurosurg. 1987;67:852-857. 3316531 10.3171/jns.1987.67.6.0852

[40] Lesueur P , Kao W, Leconte A, et al. Stereotactic radiotherapy on brain metastases with recent hemorrhagic signal: STEREO-HBM, a two-step phase 2 trial. BMC Cancer. 2020; 20:147. 32087691 10.1186/s12885-020-6569-1 PMC7036220

