# Supplementary material for: Outcomes of patients with melanoma brain metastases treated with ipilimumab and nivolumab with or without upfront comprehensive stereotactic radiosurgery
Source: Neurooncol Adv. 2026 Jan 7;8(1):vdaf276. doi: 10.1093/noajnl/vdaf276 (PMC12883207; doi:10.1093/noajnl/vdaf276)
Supplement: vdaf276_Supplementary_Data [file vdaf276_supplementary_data.docx]

Included, *n* = 165

Patients with newly diagnosed MBM started on ipi/nivo between January 1, 2018, and December 31, 2023

Excluded, *n* = 33

- Additional systemic therapy agent(s) received concomitantly with the start of ipi/nivo, *n* = 6
- No follow-up intracranial imaging, *n* = 12
- Leptomeningeal disease at MBM diagnosis, *n* = 2
- Upfront MBM therapy included WBRT, *n* = 7
- Baseline MBM count > 15, *n* = 6

Evaluable, *n* = 132

- Received upfront cSRS, *n* = 69
- Did not receive upfront cSRS, *n* = 63

**Supplemental Figure 1.** Flow diagram representing inclusion and exclusion criteria. Abbreviations: MBM, melanoma brain metastasis; ipi/nivo, ipilimumab and nivolumab; WBRT, whole brain radiatiotherapy; cSRS, comprehensive stereotactic radiosurgery.


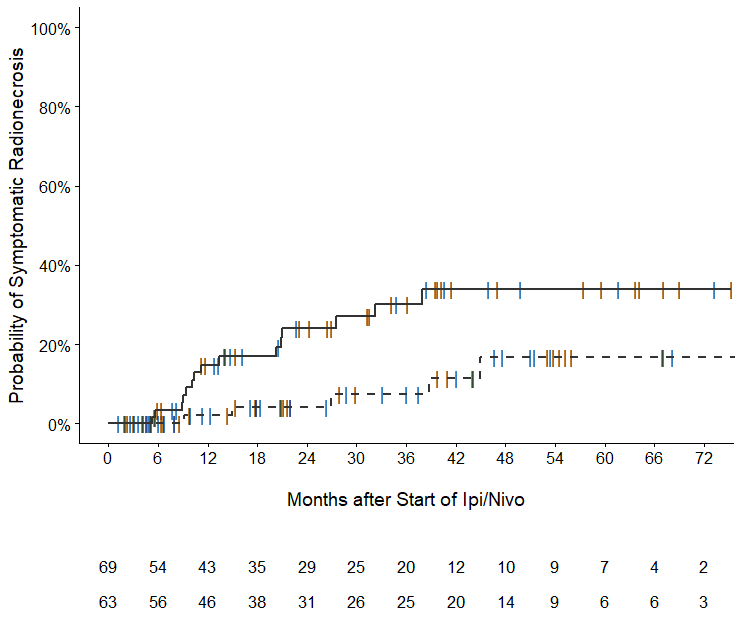


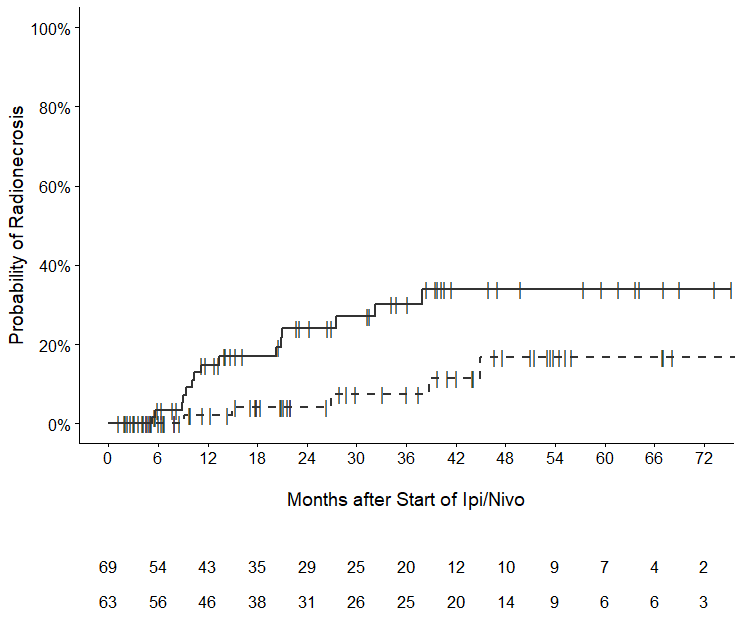

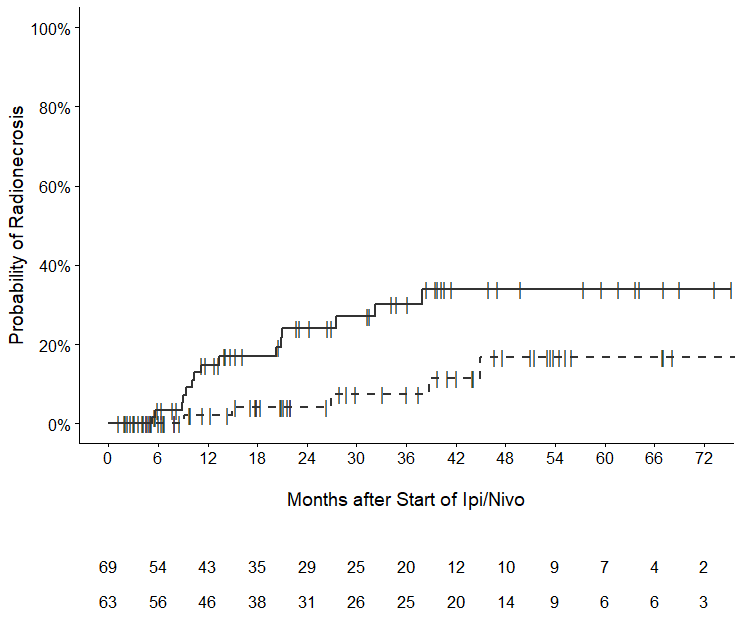


7.4% (0.0%-15.4%)

30.2% (15.0%-42.7%)

4.2% (0.0%-9.8%)

2.0% (0.0%-5.8%)

24.2% (11.0%-35.4%)

Received cSRS

**12 mo RN rate**

**24 mo RN rate**

Did not receive cSRS

**36 mo RN rate**

14.8% (4.8%-23.8%)

Number at risk:

Received cSRS

Did not receive cSRS

**Supplemental Figure 2.** Cumulative incidence of symptomatic radionecrosis among patients started on ipi/nivo for melanoma brain metastasis with and without upfront cSRS. Rates include symptomatic radionecrosis from upfront and salvage SRS courses. All estimates include 95% confidence intervals in parentheses. Abbreviations: cSRS, comprehensive stereotactic radiosurgery; ipi/nivo, ipilimumab and nivolumab; RN, radionecrosis.

**Supplemental Table 1.** Cause of death categories for patients treated with ipi/nivo for melanoma brain metastasis with and without upfront cSRS

|  | Received upfront cSRS | | | Did not receive upfront cSRS | | |
| --- | --- | --- | --- | --- | --- | --- |
|  | Count | Percentage based on number of patients  (n = 69) | Percentage based on number of deaths  (n = 32) | Count | Percentage based on number of patients  (n = 63) | Percentage based on number of deaths  (n = 26) |
| Neurologic | 10 | 14.5% | 31.3% | 11 | 17.5% | 42.3% |
| Systemic | 10 | 14.5% | 31.3% | 7 | 11.1% | 26.9% |
| Unknown | 12 | 17.4% | 37.5% | 8 | 12.7% | 30.8% |

Abbreviations: ipi/nivo, ipilimumab and nivolumab; cSRS, comprehensive stereotactic radiosurgery
